# Supplementary material for: A topological fluctuation theorem
Source: Nat Commun. 2022 May 31;13:3036. doi: 10.1038/s41467-022-30644-6 (PMC9156749; doi:10.1038/s41467-022-30644-6)
Supplement: Supplementary file 1 — Supplementary Information [file 41467_2022_30644_MOESM1_ESM.pdf]

Supplementary Material on "A topological fluctuation theorem"

Benoît Mahault,[1] Evelyn Tang,[1] and Ramin Golestanian[1, 2, *]
[1] *Max Planck Institute for Dynamics and Self-Organization, 37077 Göttingen, Germany*
[2] *Rudolf Peierls Centre for Theoretical Physics, University of Oxford, Oxford OX1 3PU, United Kingdom*

I. THE EXACT WINDING NUMBER DISTRIBUTION FOR A SINGLE VORTEX.

Here we provide the derivation of the exact winding number distribution in the case where the force-field is generated by a single vortex line. The Fokker-Planck equation associated to the dynamics of a stochastic particle in the vortex field defined by Eq. (11) of the main text is given by

$$\partial_t \mathcal{P}(r,\phi,z,t) + \frac{\gamma D}{2\pi r^2}\partial_\phi \mathcal{P}(r,\phi,z,t) = D\left[\partial^2_{rr} + \frac{1}{r}\partial_r + \frac{1}{r^2}\partial^2_{\phi\phi} + \partial^2_{zz}\right]\mathcal{P}(r,\phi,z,t), \tag{S1}$$

where at $t=0$ the distribution $\mathcal{P}(r,\phi,z,t)$ satisfies $\mathcal{P}(r,\phi,z,0) = r^{-1}\delta(r-r_0)\delta(\phi-\phi_0)\delta(z-z_0)$ with $r_0>0$, while ϕ_0 and z_0 are set to 0 without loss of generality. Due to the translational invariance of the problem along the z axis, solutions of the Fokker-Planck equation [Eq. (S1)] are written as

$$\mathcal{P}(r,\phi,z,t) = \mathcal{P}_{\rm 2D}(r,\phi,t) \times \frac{1}{\sqrt{4\pi Dt}}\exp\left(-\frac{z^2}{4Dt}\right), \tag{S2}$$

where $\mathcal{P}_{\rm 2D}(r,\phi,t)$ can itself be decomposed into separable functions of the form $e^{iu\phi}e^{-D\lambda^2 t}\rho(r)$ [1]. Meanwhile, $\rho(r)$ satisfies

$$\rho''(r) + \frac{1}{r}\rho'(r) + \left(\lambda^2 - \frac{k_u^2}{r^2}\right)\rho(r) = 0 \quad \text{with} \quad k_u^2 = u^2 + \frac{iu\gamma}{2\pi}. \tag{S3}$$

For what follows, we shall consider solutions for which ${\rm Re}\,(k_u) \geq 0$. Solutions of Eq. (S3) take the general form

$$\rho(r) = C_J(\lambda,u)J_{k_u}(\lambda r) + C_Y(\lambda,u)Y_{k_u}(\lambda r), \tag{S4}$$

where J_ν and Y_ν are Bessel functions of the first and second kind respectively, of order ν.

Moreover, since for a vortex-generated field in open space the Brownian particle avoids the origin with probability 1 [2], the distribution satisfies at all times, angles and z, $\mathcal{P}(0,\phi,z,t) = \mathcal{P}(r\to+\infty,\phi,z,t) = 0$. From this constraint, C_Y can be set to 0 in what follows. The distribution then becomes

$$\mathcal{P}_{\rm 2D}(r,\phi,t) = \int_{-\infty}^{\infty}{\rm d}u\int_0^\infty {\rm d}\lambda\, C_J(\lambda,u)J_{k_u}(\lambda r)\,e^{iu\phi}e^{-D\lambda^2 t}. \tag{S5}$$

In order to satisfy the initial condition $\mathcal{P}_{\rm 2D}(r,\phi,0) = r^{-1}\delta(r-r_0)\delta(\phi)$, we use a closure relation for Bessel functions $\int_0^\infty {\rm d}x\, xJ_\nu(xs)J_\nu(xv) = s^{-1}\delta(s-v)$, to obtain

$$\mathcal{P}_{\rm 2D}(r,\phi,t) = \frac{1}{2\pi}\int_{-\infty}^{\infty}{\rm d}u\int_0^\infty {\rm d}\lambda\,\lambda J_{k_u}(\lambda r_0)\,J_{k_u}(\lambda r)\,e^{iu\phi}e^{-D\lambda^2 t}. \tag{S6}$$

To simplify this further, we use the following relation [3]

$$\int_0^\infty {\rm d}t\, tJ_\nu(\alpha t)J_\nu(\beta t)e^{-p^2t^2} = \frac{1}{2p^2}e^{-\frac{\alpha^2+\beta^2}{4p^2}}I_\nu\left(\frac{\alpha\beta}{2p^2}\right) \tag{S7}$$

valid for $\alpha,\beta>0$, ${\rm Re}(\nu) > -1$, and where I_ν is the modified Bessel function of the first kind of order ν. This gives us

$$\mathcal{P}_{\rm 2D}(r,\phi,t) = \frac{e^{-\frac{r^2+r_0^2}{4Dt}}}{4\pi Dt}\int_{-\infty}^{\infty}{\rm d}u\, e^{iu\phi}I_{k_u}\left(\frac{r_0 r}{2Dt}\right), \tag{S8}$$

where I_ν is the modified Bessel function of the first kind, of order ν. For closed trajectories, the winding number distribution associated with a particular initial position $(r_0,\phi_0=0,z_0=0)$ is $p(n,t|r_0) \equiv \frac{1}{N}\mathcal{P}(r_0,\phi=2\pi n,0,t)$ with $N \equiv \sum_{n=-\infty}^{+\infty}p(n,t|r_0)$, giving Eq. (12) of the main text.

* ramin.golestanian@ds.mpg.de

II. TOPOLOGICAL PHASES IN ONE DIMENSION

Here we consider a one dimensional variant of the model studied in the main text and show that it allows to derive similar results. For simplicity, we examine a dynamics with two internal states A and B and where the possible transitions at site x read

$$(x)_{\mathrm{A}} \xrightarrow{\gamma_{\mathrm{ext}}} (x+a)_{\mathrm{A}}, \qquad (x)_{\mathrm{A}} \xrightarrow{\gamma'_{\mathrm{ext}}} (x-a)_{\mathrm{B}}, \qquad (x)_{\mathrm{B}} \xrightarrow{\gamma_{\mathrm{in}}} (x)_{\mathrm{A}}, \tag{S9}$$

with a denoting the lattice step while γ_{ext}, γ'_{ext} and γ_{in} are the rates between sites and internal states, respectively. The topology described by the transitions in Eq. (S9) is similar, for instance, to that studied in Refs. [4–6]. Denoting $\rho_{\mathrm{A,B}}(x,t)$ the densities at site x associated with states A and B at time t, they obey the following master equations:

$$\partial_t \rho_{\mathrm{A}}(x,t) = \gamma_{\mathrm{ext}}\left[\rho_{\mathrm{A}}(x-a,t) - \rho_{\mathrm{A}}(x,t)\right] - \gamma'_{\mathrm{ext}}\rho_{\mathrm{A}}(x,t) + \gamma_{\mathrm{in}}\rho_{\mathrm{B}}(x,t), \tag{S10a}$$

$$\partial_t \rho_{\mathrm{B}}(x,t) = \gamma'_{\mathrm{ext}}\rho_{\mathrm{A}}(x+a,t) - \gamma_{\mathrm{in}}\rho_{\mathrm{B}}(x,t). \tag{S10b}$$

Following the calculation steps described in Methods, we now define

$$\rho(x,t) \equiv \rho_{\mathrm{A}}(x,t) + \rho_{\mathrm{B}}(x,t), \qquad \delta(x,t) \equiv \rho_{\mathrm{A}}(x,t) - \rho_{\mathrm{B}}(x,t), \tag{S11}$$

as respectively the total density and the difference between the two states occupations at site x. Considering the continuous limit ($\rho(x \pm a, t) = [1 \pm a\partial_x + \frac{a^2}{2}\partial^2_{xx}]\rho + \mathcal{O}(a^3)$), we get

$$\partial_t \rho = \left[-\frac{\gamma_{\mathrm{ext}} - \gamma'_{\mathrm{ext}}}{2} a\partial_x + \frac{\gamma_{\mathrm{ext}} + \gamma'_{\mathrm{ext}}}{4} a^2 \partial^2_{xx}\right](\rho + \delta), \tag{S12a}$$

$$\partial_t \delta = \left[-\frac{\gamma_{\mathrm{ext}} + \gamma'_{\mathrm{ext}}}{2} a\partial_x + \frac{\gamma_{\mathrm{ext}} - \gamma'_{\mathrm{ext}}}{4} a^2 \partial^2_{xx}\right](n + \delta) + (\gamma_{\mathrm{in}} - \gamma'_{\mathrm{ext}})n - (\gamma_{\mathrm{in}} + \gamma'_{\mathrm{ext}})\delta. \tag{S12b}$$

It is clear from Eqs. (S12) that, contrary to δ, ρ is slow as it is conserved. Therefore, in the long-time limit and for slowly varying fields δ can be enslaved to ρ. Setting $\partial_t \delta = 0$, solving Eq. (S12b) recursively and replacing the expression of δ so obtained we find that at order a^2 Eq. (S12a) takes the simple drift-diffusion form

$$\partial_t \rho = -v\partial_x \rho + D\partial^2_{xx}\rho, \tag{S13}$$

with effective drift and diffusivity

$$v \equiv \frac{\gamma_{\mathrm{in}}(\gamma_{\mathrm{ext}} - \gamma'_{\mathrm{ext}})}{\gamma_{\mathrm{in}} + \gamma'_{\mathrm{ext}}} a, \qquad D \equiv \frac{\gamma_{\mathrm{in}}(\gamma_{\mathrm{ext}} + \gamma'_{\mathrm{ext}})(\gamma_{\mathrm{ext}} + \gamma_{\mathrm{in}})}{2(\gamma_{\mathrm{in}} + \gamma'_{\mathrm{ext}})^2} a^2.$$

Eq. (S13) thus maps the lattice model (S9) to the stochastic motion of a particle in one dimension with constant drift v and diffusivity D. Considering the model on a ring, i.e. with periodic boundary conditions, the topological fluctuation theorem [Eq. (8) of the main text] predicts for the winding number probability ratio

$$\frac{p(-n,t)}{p(n,t)} = \exp\left(-\frac{2N(\gamma_{\mathrm{ext}} - \gamma'_{\mathrm{ext}})(\gamma_{\mathrm{in}} + \gamma'_{\mathrm{ext}})}{(\gamma_{\mathrm{ext}} + \gamma'_{\mathrm{ext}})(\gamma_{\mathrm{ext}} + \gamma_{\mathrm{in}})} n\right), \tag{S14}$$

with N the total number of lattice sites. As for the two dimensional system, the rhs of Eq. (S14) takes a system size dependency which here can however be compensated by fine tuning the amplitude of the rates γ_{ext} and γ'_{ext}.

Considering now an open system, the stationary solution of Eq. (S13) corresponds to a density profile exponentially localized at the right (resp. left) edge of the system for $v > 0$ (resp. $v < 0$) over a characteristic lengthscale $\ell \equiv D/|v| = a(\gamma_{\mathrm{ext}} + \gamma'_{\mathrm{ext}})(\gamma_{\mathrm{ext}} + \gamma_{\mathrm{in}})/[2|\gamma_{\mathrm{ext}} - \gamma'_{\mathrm{ext}}|(\gamma_{\mathrm{in}} + \gamma'_{\mathrm{ext}})]$.

III. COARSE GRAINING OF THE LATTICE MODEL WITH SYMMETRIC RATES

Here we sketch the derivation of Eq. (15) of the main text for the case where the lattice model of Fig. 4 includes symmetric transition rates. The derivation follows similar lines as the one presented in Methods.

The bulk dynamics Let us first recall the transition rules of the lattice model. Considering a site at position (x, y) the allowed transitions are:

$$\begin{array}{ccc} (x,y)_\mathrm{D} & \rightleftarrows & (x,y)_\mathrm{A} \\ \uparrow\downarrow & \gamma_\mathrm{in}^{(\prime)} & \uparrow\downarrow \\ (x,y)_\mathrm{C} & \rightleftarrows & (x,y)_\mathrm{B} \end{array} \quad ; \quad \begin{array}{ccc} (x,y+a)_\mathrm{B} & \rightleftarrows & (x+a,y+a)_\mathrm{C} \\ \uparrow\downarrow & \gamma_\mathrm{ext}^{(\prime)} & \uparrow\downarrow \\ (x,y)_\mathrm{A} & \rightleftarrows & (x+a,y)_\mathrm{D} \end{array} \quad , \tag{S15}$$

where the letters A-D label the four internal states and a is the lattice spacing. In Eq. (S15) γ_ext and γ'_ext denote the external clockwise and counter-clockwise transition rates, while γ_in and γ'_in are the counter-clockwise and clockwise internal rates. Taking into account all possible transitions and neglecting the influence of the system's boundary, the master equations governing the bulk dynamics read

$$\begin{aligned} \partial_t\rho_{\sigma(i)}(\boldsymbol{x},t) =& \gamma_\mathrm{ext}\left[\rho_{\sigma(i-1)}(\boldsymbol{x}+\Delta\boldsymbol{x}_i,t)-\rho_{\sigma(i)}(\boldsymbol{x},t)\right]+\gamma'_\mathrm{ext}\left[\rho_{\sigma(i+1)}(\boldsymbol{x}+\Delta\boldsymbol{x}_{i-1},t)-\rho_{\sigma(i)}(\boldsymbol{x},t)\right] \\ &+\gamma_\mathrm{in}\left[\rho_{\sigma(i+1)}(\boldsymbol{x},t)-\rho_{\sigma(i)}(\boldsymbol{x},t)\right]+\gamma'_\mathrm{in}\left[\rho_{\sigma(i-1)}(\boldsymbol{x},t)-\rho_{\sigma(i)}(\boldsymbol{x},t)\right]\,,, \end{aligned} \tag{S16}$$

where, as in the main text, we have used the 4-periodic map σ: $i \in \{0,...,3\} \to \{\mathrm{A},...,\mathrm{D}\}$, as well as the notations $\boldsymbol{x} = x\hat{\boldsymbol{e}}_x + y\hat{\boldsymbol{e}}_y$ and $\Delta\boldsymbol{x}_i = a\mathcal{R}(-\frac{i\pi}{2})\hat{\boldsymbol{e}}_x$. Defining the following fields

$$\begin{aligned} \rho_\mathrm{b} &\equiv \tfrac{1}{4}\left(\rho_\mathrm{A}+\rho_\mathrm{B}+\rho_\mathrm{C}+\rho_\mathrm{D}\right), \qquad & \rho_1 &\equiv \tfrac{1}{4}\left(\rho_\mathrm{A}-\rho_\mathrm{B}+\rho_\mathrm{C}-\rho_\mathrm{D}\right), \\ \rho_2 &\equiv \tfrac{1}{4}\left(\rho_\mathrm{A}+\rho_\mathrm{B}-\rho_\mathrm{C}-\rho_\mathrm{D}\right), \qquad & \rho_3 &\equiv \tfrac{1}{4}\left(\rho_\mathrm{A}-\rho_\mathrm{B}-\rho_\mathrm{C}+\rho_\mathrm{D}\right), \end{aligned}$$

and taking the continuum limit Eqs. (S16) are recast up to order a^2 as

$$\partial_t\rho_\mathrm{b} = \frac{(\gamma_\mathrm{ext}+\gamma'_\mathrm{ext})a^2}{4}\Delta\rho_\mathrm{b} - \frac{(\gamma_\mathrm{ext}-\gamma'_\mathrm{ext})a^2}{4}\mathcal{D}\rho_1 - \frac{\gamma_\mathrm{ext}a}{2}\left(\partial\rho_2-\bar{\partial}\rho_3\right) - \frac{\gamma'_\mathrm{ext}a}{2}\left(\bar{\partial}\rho_2+\partial\rho_3\right) \tag{S17a}$$

$$\begin{aligned}\partial_t\rho_1 =& \frac{(\gamma_\mathrm{ext}-\gamma'_\mathrm{ext})a^2}{4}\mathcal{D}\rho_\mathrm{b} - \frac{(\gamma_\mathrm{ext}+\gamma'_\mathrm{ext})a^2}{4}\Delta\rho_1 - \frac{\gamma_\mathrm{ext}a}{2}\left(\bar{\partial}\rho_2-\partial\rho_3\right) + \frac{\gamma'_\mathrm{ext}a}{2}\left(\partial\rho_2+\bar{\partial}\rho_3\right) \\ &- 2(\gamma_\mathrm{ext}+\gamma'_\mathrm{ext}+\gamma_\mathrm{in}+\gamma'_\mathrm{in})\rho_1, \end{aligned} \tag{S17b}$$

$$\begin{aligned}\partial_t\rho_2 =& \frac{\gamma_\mathrm{ext}a}{2}\left(\bar{\partial}\rho_\mathrm{b}-\partial\rho_1\right) + \frac{\gamma'_\mathrm{ext}a}{2}\left(\partial\rho_\mathrm{b}+\bar{\partial}\rho_1\right) - \frac{(\gamma_\mathrm{ext}+\gamma'_\mathrm{ext})a^2}{4}\mathcal{D}\rho_2 + \frac{(\gamma_\mathrm{ext}-\gamma'_\mathrm{ext})a^2}{4}\Delta\rho_3 \\ &- (\gamma_\mathrm{ext}+\gamma'_\mathrm{ext}+\gamma_\mathrm{in}+\gamma'_\mathrm{in})\rho_2 + (\gamma_\mathrm{ext}-\gamma'_\mathrm{ext}-\gamma_\mathrm{in}+\gamma'_\mathrm{in})\rho_3, \end{aligned} \tag{S17c}$$

$$\begin{aligned}\partial_t\rho_3 =& \frac{\gamma_\mathrm{ext}a}{2}\left(\partial\rho_\mathrm{b}-\bar{\partial}\rho_1\right) - \frac{\gamma'_\mathrm{ext}a}{2}\left(\bar{\partial}\rho_\mathrm{b}+\partial\rho_1\right) - \frac{(\gamma_\mathrm{ext}-\gamma'_\mathrm{ext})a^2}{4}\Delta\rho_2 + \frac{(\gamma_\mathrm{ext}+\gamma'_\mathrm{ext})a^2}{4}\mathcal{D}\rho_3 \\ &- (\gamma_\mathrm{ext}-\gamma'_\mathrm{ext}-\gamma_\mathrm{in}+\gamma'_\mathrm{in})\rho_2 - (\gamma_\mathrm{ext}+\gamma'_\mathrm{ext}+\gamma_\mathrm{in}+\gamma'_\mathrm{in})\rho_3, \end{aligned} \tag{S17d}$$

where we have used the following definitions for the spatial derivative operators

$$\partial \equiv \partial_x + \partial_y, \qquad \bar{\partial} \equiv \partial_x - \partial_y, \qquad \Delta \equiv \partial^2_{xx} + \partial^2_{yy}, \qquad \mathcal{D} \equiv \partial^2_{xx} - \partial^2_{yy}.$$

As for the fully chiral case, ρ_b is the only slow field in Eq. (S17) and we now enslave $\rho_{1,2,3}$. Keeping terms up to order a^2, we find that $\rho_1 = \mathcal{O}(a)$ can be neglected, while

$$\begin{pmatrix} \alpha & -\beta \\ \beta & \alpha \end{pmatrix} \begin{pmatrix} \rho_2 \\ \rho_3 \end{pmatrix} = \frac{a}{2} \begin{pmatrix} \gamma_\mathrm{ext}\bar{\partial} + \gamma'_\mathrm{ext}\partial \\ \gamma_\mathrm{ext}\partial - \gamma'_\mathrm{ext}\bar{\partial} \end{pmatrix} \rho_\mathrm{b} + \mathcal{O}(a^2), \tag{S18}$$

with $\alpha \equiv \gamma_\mathrm{ext} + \gamma'_\mathrm{ext} + \gamma_\mathrm{in} + \gamma'_\mathrm{in}$ and $\beta \equiv \gamma_\mathrm{ext} - \gamma'_\mathrm{ext} - (\gamma_\mathrm{in} - \gamma'_\mathrm{in})$. Solving the above equation for $\rho_{2,3}$ and replacing the solution into Eq. (S17a) we recover

$$\partial_t\rho_\mathrm{b} = D_\mathrm{b}\Delta\rho_\mathrm{B}, \qquad \text{with } D_\mathrm{b} \equiv \frac{a^2}{4}\frac{(\gamma_\mathrm{ext}+\gamma'_\mathrm{ext})(\gamma_\mathrm{in}^2+\gamma'^2_\mathrm{in}) + (\gamma_\mathrm{in}+\gamma'_\mathrm{in})(\gamma^2_\mathrm{ext}+\gamma'^2_\mathrm{ext})}{(\gamma'_\mathrm{ext}+\gamma_\mathrm{in})^2 + (\gamma_\mathrm{ext}+\gamma'_\mathrm{in})^2}. \tag{S19}$$

As for the fully chiral case presented in Methods, we find that the bulk dynamics is fully diffusive with an isotropic bulk diffusivity symmetric by exchange of indices int and ext, while its limiting behaviors read

$$D_{\rm b} \underset{\gamma_{\rm ext},\gamma'_{\rm ext}\gg\gamma_{\rm in},\gamma'_{\rm in}}{\sim} \frac{(\gamma_{\rm in}+\gamma'_{\rm in})a^2}{4}, \qquad \text{and} \qquad D_{\rm b} \underset{\gamma_{\rm ext},\gamma'_{\rm ext}\ll\gamma_{\rm in},\gamma'_{\rm in}}{\sim} \frac{(\gamma_{\rm ext}+\gamma'_{\rm ext})a^2}{4},$$

so that it always remains finite even for fast external rates.

The dynamics near boundaries We now turn to the characterization of the dynamics at the lower edge of the system, which we couple to the bulk density $\rho_{\rm b}$ which plays the role of the density at sites A and D. The corresponding master equations read

$$\partial_t\rho_{\rm B}(x,y,t) = -\gamma_{\rm ext}\rho_{\rm B}(x,y,t) + \gamma'_{\rm ext}\rho_{\rm C}(x+a,y,t) + \gamma_{\rm in}\left[\rho_{\rm C}(x,y,t)-\rho_{\rm B}(x,y,t)\right] + \gamma'_{\rm in}\left[\rho_{\rm b}(x,y,t)-\rho_{\rm B}(x,y,t)\right], \tag{S20a}$$

$$\partial_t\rho_{\rm C}(x,y,t) = \gamma_{\rm ext}\rho_{\rm B}(x-a,y,t) - \gamma'_{\rm ext}\rho_{\rm C}(x,y,t) + \gamma_{\rm in}\left[\rho_{\rm b}(x,y,t)-\rho_{\rm C}(x,y,t)\right] + \gamma'_{\rm in}\left[\rho_{\rm B}(x,y,t)-\rho_{\rm C}(x,y,t)\right], \tag{S20b}$$

$$\partial_t\rho_{\rm b}(x,y,t) = D_{\rm b}\Delta\rho_{\rm b}(x,y,t) + \frac{\gamma_{\rm in}}{2}\left[\rho_{\rm B}(x,y,t)-\rho_{\rm b}(x,y,t)\right] + \frac{\gamma'_{\rm in}}{2}\left[\rho_{\rm C}(x,y,t)-\rho_{\rm b}(x,y,t)\right]. \tag{S20c}$$

Similarly to the fully chiral case, we define

$$\rho_{\rm e} \equiv \tfrac{1}{4}\left(2\rho_{\rm b}+\rho_{\rm B}+\rho_{\rm C}\right), \qquad \rho'_1 \equiv \tfrac{1}{4}\left(2\rho_{\rm b}+\rho_{\rm B}-\rho_{\rm C}\right), \qquad \rho'_2 \equiv \tfrac{1}{4}\left(2\rho_{\rm b}-\rho_{\rm B}+\rho_{\rm C}\right).$$

Taking the continuous limit and expanding Eqs. (S20) up to second order in a, we find after some algebra

$$\partial_t\rho_{\rm e} = -\frac{\gamma_{\rm ext}a}{2}\left(\partial_x - \frac{a}{2}\partial^2_{xx}\right)(\rho_{\rm e}-\rho'_2) + \frac{\gamma'_{\rm ext}a}{2}\left(\partial_x + \frac{a}{2}\partial^2_{xx}\right)(\rho_{\rm e}-\rho'_1) + \frac{D_{\rm b}}{2}\Delta(\rho'_1+\rho'_2), \tag{S21a}$$

$$\begin{aligned}\partial_t\rho'_1 = {}&(\gamma_{\rm in}+\gamma'_{\rm ext}-\gamma_{\rm ext})\rho_{\rm e} - (\gamma'_{\rm in}+\gamma'_{\rm ext}+\tfrac{3}{2}\gamma_{\rm in})\rho'_1 - (\tfrac{1}{2}\gamma_{\rm in}-\gamma'_{\rm in}-\gamma_{\rm ext})\rho'_2\\ &+\frac{\gamma_{\rm ext}a}{2}\partial_x(\rho_{\rm e}-\rho'_2) + \frac{\gamma'_{\rm ext}a}{2}\partial_x(\rho_{\rm e}-\rho'_1) + \mathcal{O}(a^2),\end{aligned} \tag{S21b}$$

$$\begin{aligned}\partial_t\rho'_2 = {}&(\gamma_{\rm ext}+\gamma'_{\rm in}-\gamma'_{\rm ext})\rho_{\rm e} + (\gamma_{\rm in}+\gamma'_{\rm ext}-\tfrac{1}{2}\gamma'_{\rm in})\rho'_1 - (\gamma_{\rm ext}+\gamma_{\rm in}+\tfrac{3}{2}\gamma'_{\rm in})\rho'_2\\ &-\frac{\gamma_{\rm ext}a}{2}\partial_x(\rho_{\rm e}-\rho'_2) - \frac{\gamma'_{\rm ext}a}{2}\partial_x(\rho_{\rm e}-\rho'_1) + \mathcal{O}(a^2).\end{aligned} \tag{S21c}$$

Solving the above two last equations for $\rho'_{1,2}$ at zeroth order in a, we get the lengthly expressions

$$\rho'^{(0)}_1 = \frac{\gamma'_{\rm in}(2\gamma'_{\rm in}+2\gamma_{\rm in}+\gamma_{\rm ext}+\gamma'_{\rm ext}) + \gamma_{\rm in}(2\gamma_{\rm in}+3\gamma'_{\rm ext}-\gamma_{\rm ext})}{\gamma'_{\rm in}(4\gamma'_{\rm in}+4\gamma_{\rm in}+3\gamma_{\rm ext}+\gamma'_{\rm ext}) + \gamma_{\rm in}(4\gamma_{\rm in}+3\gamma'_{\rm ext}+\gamma_{\rm ext})}\rho_{\rm e} \equiv \alpha_1\rho_{\rm e},$$

$$\rho'^{(0)}_2 = \frac{\gamma'_{\rm in}(2\gamma'_{\rm in}+2\gamma_{\rm in}+3\gamma_{\rm ext}-\gamma'_{\rm ext}) + \gamma_{\rm in}(2\gamma_{\rm in}+\gamma'_{\rm ext}+\gamma_{\rm ext})}{\gamma'_{\rm in}(4\gamma'_{\rm in}+4\gamma_{\rm in}+3\gamma_{\rm ext}+\gamma'_{\rm ext}) + \gamma_{\rm in}(4\gamma_{\rm in}+3\gamma'_{\rm ext}+\gamma_{\rm ext})}\rho_{\rm e} \equiv \alpha_2\rho_{\rm e},$$

which imply the following relative occupations for the boundary states in the limit of vanishing gradients

$$\frac{\rho_{\rm B}}{\rho_{\rm b}} = 1 - \frac{\gamma'_{\rm in}(\gamma_{\rm ext}-\gamma'_{\rm ext})}{\gamma_{\rm in}(\gamma_{\rm in}+\gamma'_{\rm ext}) + \gamma'_{\rm in}(\gamma'_{\rm in}+\gamma_{\rm in}+\gamma_{\rm ext})}, \qquad \frac{\rho_{\rm C}}{\rho_{\rm b}} = 1 + \frac{\gamma_{\rm in}(\gamma_{\rm ext}-\gamma'_{\rm ext})}{\gamma_{\rm in}(\gamma_{\rm in}+\gamma'_{\rm ext}) + \gamma'_{\rm in}(\gamma'_{\rm in}+\gamma_{\rm in}+\gamma_{\rm ext})}.$$

Using the definition of $\rho_{\rm e}$, we thus obtain the ratio of edge to bulk densities

$$\frac{\rho_{\rm e}}{\rho_{\rm b}} = 1 + \frac{1}{4}\frac{(\gamma_{\rm in}-\gamma'_{\rm in})(\gamma_{\rm ext}-\gamma'_{\rm ext})}{\gamma_{\rm in}(\gamma_{\rm in}+\gamma'_{\rm ext}) + \gamma'_{\rm in}(\gamma'_{\rm in}+\gamma_{\rm in}+\gamma_{\rm ext})}, \tag{S22}$$

whose expression for $\gamma_{\rm in},\gamma'_{\rm in} \ll \gamma_{\rm ext},\gamma'_{\rm ext}$ is reported in Eq. (16) of the main text. In the opposite limit of fast internal rates, Eq. (S22) reads

$$\frac{\rho_{\rm e}}{\rho_{\rm b}} \underset{\gamma_{\rm in},\gamma'_{\rm in}\gg\gamma_{\rm ext},\gamma'_{\rm ext}}{\sim} 1 + \frac{1}{4}\frac{\gamma_{\rm ext}}{\gamma_{\rm in}}\frac{(1-\zeta_{\rm in})(1-\zeta_{\rm ext})}{1+\zeta_{\rm in}+\zeta^2_{\rm in}}, \tag{S23}$$

where we have used the definitions $\zeta_{\rm in} = \gamma'_{\rm in}/\gamma_{\rm in}$ and $\zeta_{\rm ext} = \gamma'_{\rm ext}/\gamma_{\rm ext}$. In contrast with the fast external rate limit, Eq. (S23) implies no significant density accumulation at the system boundary.

Using the expressions of $\rho'_{1,2}$ obtained at zeroth order in a, we now evaluate the linear order contribution as

$$\rho_1'^{(1)} = \frac{a(3\gamma_{\rm in}+\gamma'_{\rm in})[(\gamma_{\rm ext}+\gamma'_{\rm ext})(\gamma_{\rm in}^2+\gamma_{\rm in}\gamma'_{\rm in}+\gamma'^2_{\rm in})+2\gamma_{\rm ext}\gamma'_{\rm ext}(\gamma_{\rm in}+\gamma'_{\rm in})]}{[\gamma'_{\rm in}(3\gamma_{\rm ext}+\gamma'_{\rm ext}+4\gamma_{\rm in}+4\gamma'_{\rm in})+\gamma_{\rm in}(\gamma_{\rm ext}+3\gamma'_{\rm ext}+4\gamma_{\rm in})]^2}\partial_x\rho_{\rm e}, \qquad \rho_2'^{(1)} = -\frac{\gamma_{\rm in}+3\gamma'_{\rm in}}{\gamma'_{\rm in}+3\gamma_{\rm in}}\rho_1^{(1)}.$$

Replacing the expressions of $\rho'_{1,2}$ as function of $\rho_{\rm e}$ in Eq. (S21a), we recover Eq. (15) of the main text with the following coefficients

$$\begin{aligned}
v_\parallel &= \frac{a(\gamma_{\rm ext}-\gamma'_{\rm ext})(\gamma_{\rm in}^2+\gamma_{\rm in}\gamma'_{\rm in}+\gamma'^2_{\rm in})}{\gamma'_{\rm in}(3\gamma_{\rm ext}+\gamma'_{\rm ext}+4\gamma_{\rm in}+4\gamma'_{\rm in})+\gamma_{\rm in}(\gamma_{\rm ext}+3\gamma'_{\rm ext}+4\gamma_{\rm in})},\\
D_\perp &= \frac{2D_{\rm b}((\gamma'_{\rm ext}+\gamma_{\rm in})\gamma_{\rm in}+(\gamma_{\rm in}+\gamma_{\rm ext})\gamma'_{\rm in}+\gamma'^2_{\rm in})}{\gamma'_{\rm in}(3\gamma_{\rm ext}+\gamma'_{\rm ext}+4\gamma_{\rm in}+4\gamma'_{\rm in})+\gamma_{\rm in}(\gamma_{\rm ext}+3\gamma'_{\rm ext}+4\gamma_{\rm in})},\\
D_\parallel &= \frac{2a^2(\gamma_{\rm in}^2+\gamma_{\rm in}\gamma'_{\rm in}+\gamma'^2_{\rm in})[(\gamma_{\rm ext}+\gamma'_{\rm ext})(\gamma_{\rm in}^2+\gamma_{\rm in}\gamma'_{\rm in}+\gamma'^2_{\rm in})+2\gamma_{\rm ext}\gamma'_{\rm ext}(\gamma_{\rm in}+\gamma'_{\rm in})]}{[\gamma'_{\rm in}(3\gamma_{\rm ext}+\gamma'_{\rm ext}+4\gamma_{\rm in}+4\gamma'_{\rm in})+\gamma_{\rm in}(\gamma_{\rm ext}+3\gamma'_{\rm ext}+4\gamma_{\rm in})]^2}+D_\perp.
\end{aligned}$$

Taking the limit $\gamma_{\rm in},\gamma'_{\rm in}\gg\gamma_{\rm ext},\gamma'_{\rm ext}$, these coefficients simply as

$$v_\parallel \sim \frac{a(\gamma_{\rm ext}-\gamma'_{\rm ext})}{4}, \qquad D_\perp \sim \frac{a^2(\gamma_{\rm ext}+\gamma'_{\rm ext})}{8}, \qquad D_\parallel \sim \frac{5a^2(\gamma_{\rm ext}+\gamma'_{\rm ext})}{8},$$

which resemble those expected for normal diffusion on a square lattice. In the opposite limit of fast external rates, we conversely find

$$\begin{aligned}
v_\parallel &\sim a\gamma_{\rm in}\frac{(1-\zeta_{\rm ext})(1+\zeta_{\rm in}+\zeta_{\rm in}^2)}{1+3\zeta_{\rm in}+\zeta_{\rm ext}(3+\zeta_{\rm in})},\\
D_\perp &\sim \frac{a^2\gamma_{\rm in}}{2}\frac{(1+\zeta_{\rm in})(\zeta_{\rm in}+\zeta_{\rm ext})}{1+3\zeta_{\rm in}+\zeta_{\rm ext}(3+\zeta_{\rm in})},\\
D_\parallel &\sim 4a^2\gamma_{\rm in}\frac{\zeta_{\rm ext}(1+\zeta_{\rm in})(1+\zeta_{\rm in}+\zeta_{\rm in}^2)}{[1+3\zeta_{\rm in}+\zeta_{\rm ext}(3+\zeta_{\rm in})]^2}+D_\perp.
\end{aligned}$$

In the limit of strong chirality $\zeta_{\rm ext},\zeta_{\rm in}\to 0$, expressing these coefficients at leading order in $\zeta_{\rm ext}$ and $\zeta_{\rm ext}$ leads to the expressions given in the main text.

SUPPLEMENTARY REFERENCES

[1] H. Wen and J.-L. Thiffeault, Winding of a brownian particle around a point vortex, Philosophical Transactions of the Royal Society A: Mathematical, Physical and Engineering Sciences **377**, 20180347 (2019).
[2] F. Spitzer, Some theorems concerning 2-dimensional brownian motion, Transactions of the American Mathematical Society **87**, 187 (1958).
[3] I. S. Gradshteyn and I. M. Ryzhik, *Table of integrals, series, and products*, seventh ed. (Elsevier/Academic Press, Amsterdam, 2007) pp. xlviii+1171.
[4] A. Murugan and S. Vaikuntanathan, Topologically protected modes in non-equilibrium stochastic systems, Nat. Commun. **8**, 13881 (2017).
[5] E. Tang, J. Agudo-Canalejo, and R. Golestanian, Topology protects chiral edge currents in stochastic systems, Phys. Rev. X **11**, 031015 (2021).
[6] J. Knebel, P. M. Geiger, and E. Frey, Topological phase transition in coupled rock-paper-scissors cycles, Phys. Rev. Lett. **125**, 258301 (2020).
